# Supplementary material for: Towards unified AI-driven fracture mechanics: the extended deep energy method (XDEM)
Source: Nat Commun. 2026 Aug 15;17:8492. doi: 10.1038/s41467-026-76748-1 (PMC13484471; doi:10.1038/s41467-026-76748-1)
Supplement: Supplementary file 3 [file 41467_2026_76748_MOESM3_ESM.zip › pic/Results/Stress_intensity_factor/Cross/XDEM/angle_30/crack_detailed_fields.pdf]

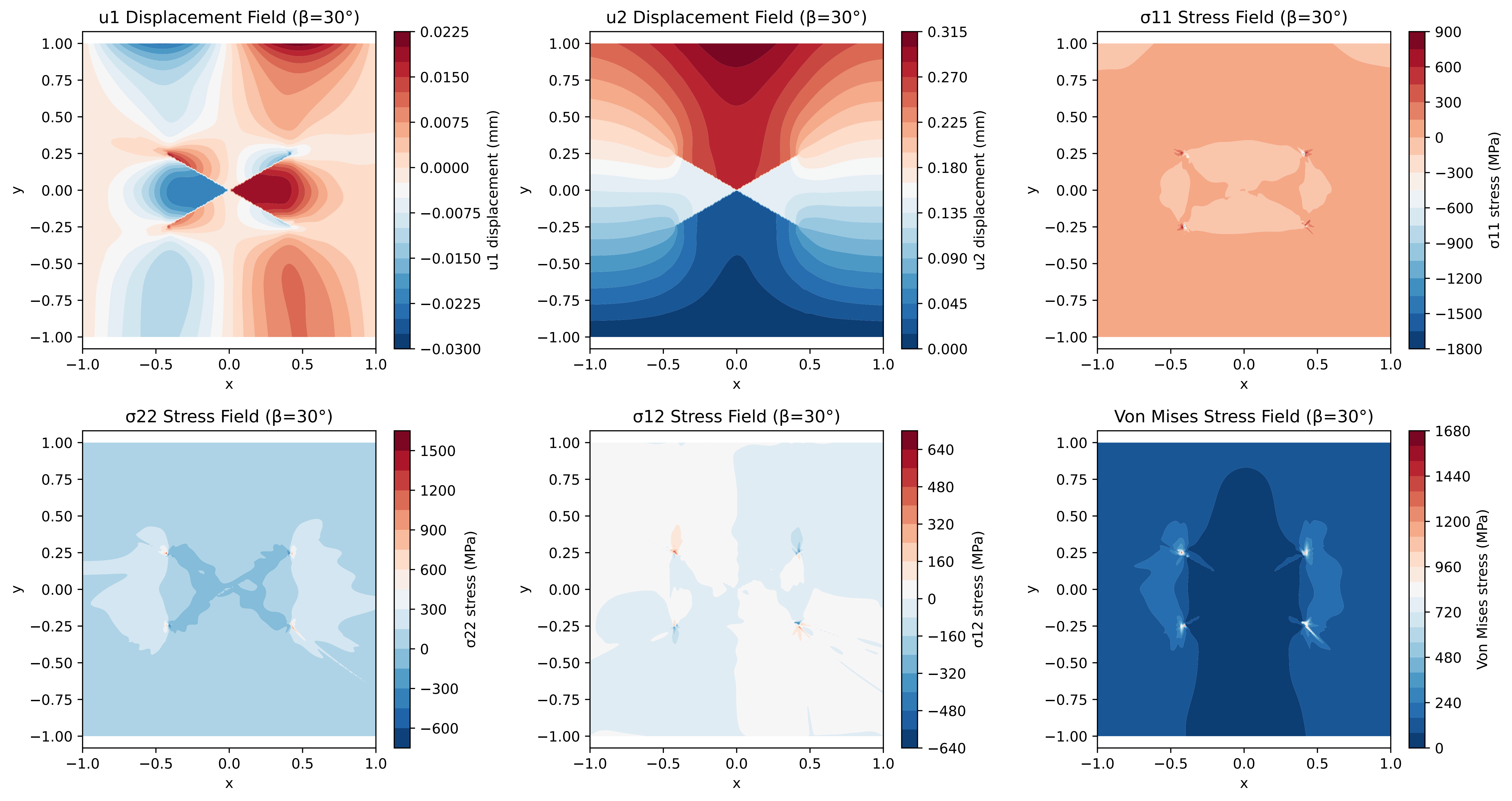

u1 Displacement Field (β=30°)
u1 displacement (mm)
0.0225
0.0150
0.0075
0.0000
−0.0075
−0.0150
−0.0225
−0.0300
u2 Displacement Field (β=30°)
u2 displacement (mm)
0.315
0.270
0.225
0.180
0.135
0.090
0.045
0.000
σ11 Stress Field (β=30°)
σ11 stress (MPa)
900
600
300
0
−300
−600
−900
−1200
−1500
−1800
σ22 Stress Field (β=30°)
σ22 stress (MPa)
1500
1200
900
600
300
0
−300
−600
σ12 Stress Field (β=30°)
σ12 stress (MPa)
640
480
320
160
0
−160
−320
−480
−640
Von Mises Stress Field (β=30°)
Von Mises stress (MPa)
1680
1440
1200
960
720
480
240
0
x
y
−1.0
−0.5
0.0
0.5
1.0
1.00
0.75
0.50
0.25
0.00
−0.25
−0.50
−0.75
−1.00
